# Efficient and reliable methods for estimating the abundance of keystone coastal macrofauna over large spatial scales

**DOI:** 10.1002/ece3.70088

**Published:** 2024-08-13

**Authors:** Molly Reamon, Johanna B. Marcussen, Ane T. Laugen, Lars M. Korslund

**Affiliations:** ^1^ Centre for Coastal Research, Department of Natural Sciences University of Agder Kristiansand Norway; ^2^ Institute of Marine Research Tromsø Norway

**Keywords:** blue mussel, European flat oyster, marine habitat mapping, Pacific oyster, towed video, underwater imagery

## Abstract

Coastal bivalves are important ecosystem engineers, and identifying critical habitats can enhance conservation outcomes for threated keystone species as well as determining hotspots for invasive species. As early action is more efficient in both conservation and mitigation of species invasions, efficient and reliable tools for mapping and monitoring species over large scales are essential. We assessed the reliability and efficiency of towed video and quadrat sampling for estimating the abundance of three keystone macrofaunal bivalve species. To assess reliability, we compared the measured density based on each of the two methods to the “true” density estimated by manually surveying an entire transect. We found that both the video and quadrat method caused underestimation of the density of bivalves, but that the amount of underestimation was comparable, and further that both methods took substantially less time than surveying an entire transect manually. The video method underestimated the abundance of Pacific oysters (*Magallana gigas*), European flat oysters (*Ostrea edulis*), and blue mussels (*Mytilus* spp.) by 23%, 24%, and 16%, respectively. The causes of underestimation for the two oyster species were bivalves grouped in clusters, large amounts of small individuals, and generally higher abundances. While *Mytilus* spp. were underestimated overall, here observer experience was important, with inexperienced observers overestimating and experienced observers underestimating. Our study found both methods to be reliable and efficient for estimating the abundance of three keystone macrofaunal species, suggesting their potential applicability to other sessile or slow‐moving species. We propose that these methods, due to their efficiency, can advance scientific knowledge and enhance conservation outcomes by establishing population baselines, assessing trends over time, and identifying and protecting critical habitats.

## Graphical abstract

We assess the reliability and efficiency of towed video and quadrat sampling for estimating the abundance of three keystone macrofauna species. To assess reliability, we compared the observed density estimates from each method with in situ data and calculated the resulting error for each method. We found both methods to be reliable and efficient and propose that they can enhance conservation outcomes by establishing population baselines, assessing trends over time, and identifying and protecting critical habitats.

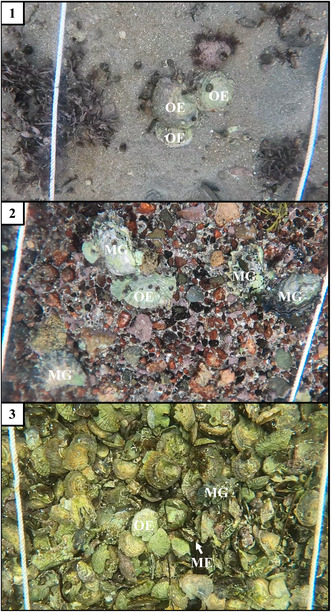

## 1 INTRODUCTION

Coastal sessile species, such as bivalves, are impacted by a myriad of anthropogenic factors, such as habitat degradation, climate change, competition, diseases from invasive species, and pollution (Beck et al., 2009; Gibson et al., 2007). The aforementioned factors, alone and combined, often lead to shifts in species distributions and thereby changes in local ecosystem dynamics (Mainka & Howard, 2010; Rahel & Olden, 2008; Stachowicz et al., 2002). Being able to predict and detect such range shifts is crucial for prioritization of action aiming to protect critical ecosystems or for early mitigation of spread of invasive species (Simberloff et al., 2013). Therefore, developing reliable and cost‐effective methods for mapping and monitoring species over large spatial areas will—in the face of limited resources—not only assist with evidence‐based prioritization of management actions, but also boost ecological research (Renn et al., 2024).

**FIGURE 1:**
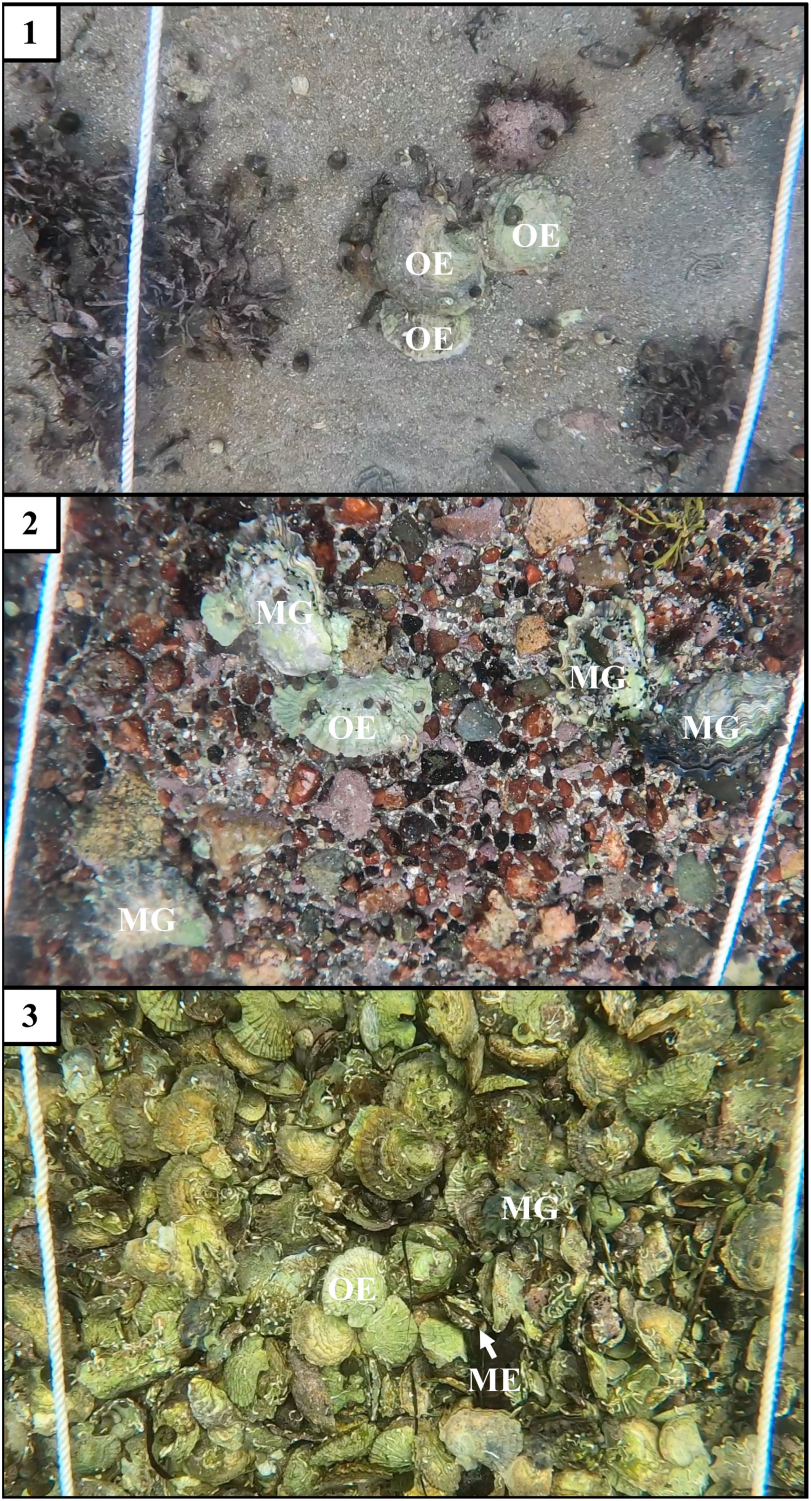
A still image from each of the three sites showing examples of how *Magallana gigas* (MG), *Ostrea edulis* (OE), and *Mytilus* spp. (ME) look in the videos.

**FIGURE 2:**
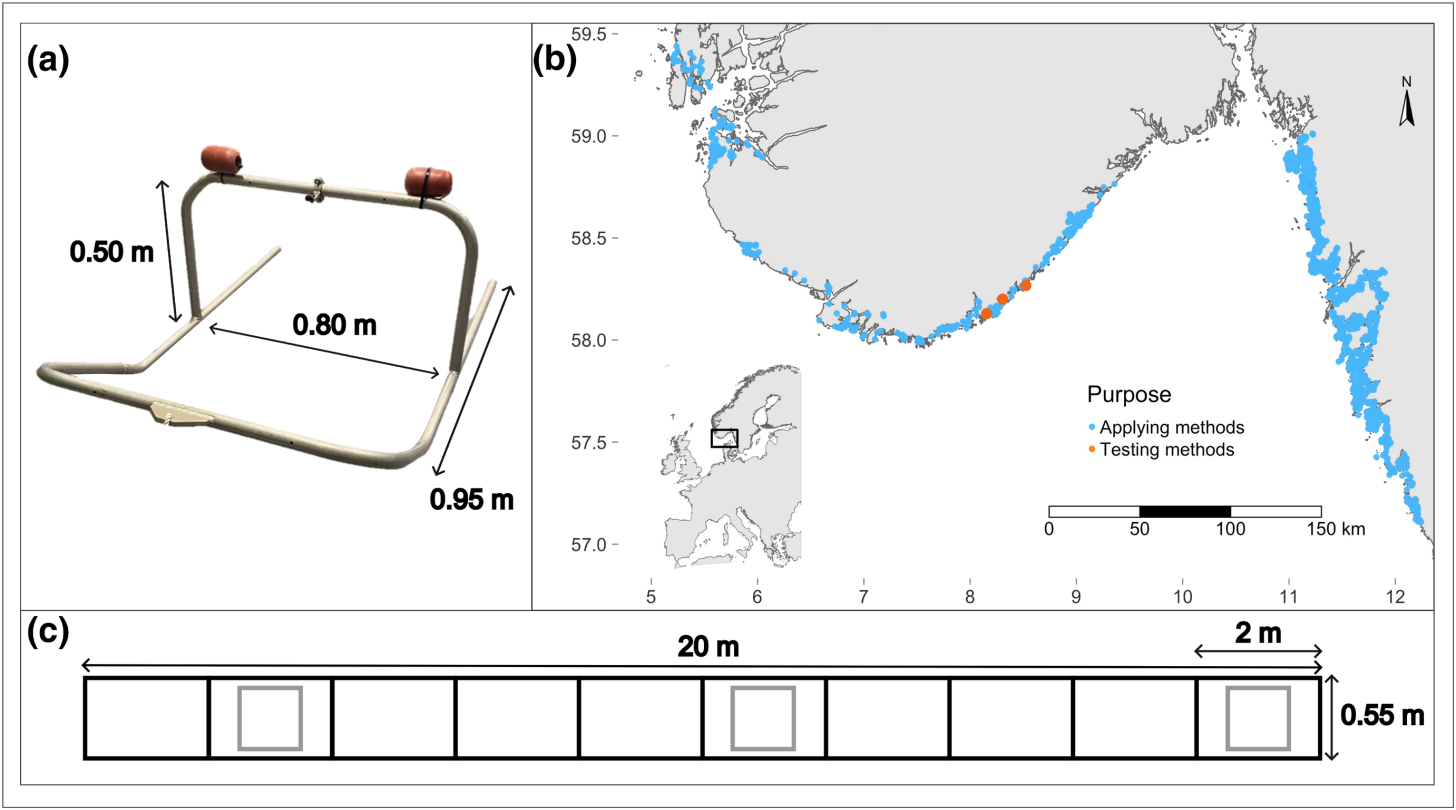
Photo of the towed video sled and its dimensions, in which the GoPro camera is attached at the top (a), map of the sites where the towed video and quadrat method have been tested in this study (three sites; orange) and applied in the field in other studies (blue) (map created using the *tmap* package v3.3–4, Tennekes, 2018); (b), and a schematic drawing of the dimensions of the rope ladder used to sample bivalves in situ (black) with the three metal squares (gray) placed within the rope ladder (c).

Underwater video techniques have emerged as efficient tools for mapping large spatial areas in a non‐invasive way (Mallet & Pelletier, 2014). Seabed towed video (hereafter towed video) is a method used to survey benthic and epifaunal species by towing a sled, mounted with a camera, behind a boat while the sled maintains contact with the seabed (Figures 1 and 2a; Mallet & Pelletier, 2014; Sheehan et al., 2016; Spencer et al., 2005; Thorngren et al., 2017). Towed video faces challenges in surveying mobile species that are sensitive to boat noise or disturbed by the movement of the video sled itself (Mallet & Pelletier, 2014; McIntyre et al., 2015), but on the other hand, towed video is a suitable method for surveying sessile epibenthic species, such as certain bivalve species.

Towed video has, in recent years, been used to estimate the abundance and map the distribution of coastal bivalves along Norwegian and Swedish coastlines (Bergström et al., 2021, 2022; Reamon et al., 2022; Thorngren et al., 2019; Figure 2b). However, despite it being commonly used, this method has not been sufficiently evaluated. First, the detectability of individuals depends on the density they occur in (Thorngren et al., 2017), and may also depend on a species’ tendency to form three‐dimensional reef structures, the amount of algae present, and the substrate characteristics of the site. Indeed, towed video has been found to underestimate the abundance of European flat oysters (*Ostrea edulis*), partially explained by individuals forming clusters or being covered by sediment or algae (Thorngren et al., 2017). It is therefore necessary to assess the error from towed video across several species. Second, towed video cannot be used for sampling in the shallowest habitats as the camera must be submerged and the field of view becomes more limited the closer the camera is to the seabed. It is therefore necessary to develop a method to survey shallow waters that is as efficient as, and yields comparable results to, towed video. Third, it is unknown how efficient the towed video method is in terms of time spent compared to other established methods.

The overall aim of this study is to test the reliability and efficiency of two commonly used methods for estimating the abundance of epibenthic macrofauna. We focus on three ecologically important coastal bivalve species: the European flat oyster (*Ostrea edulis*), the Pacific oyster (*Magallana gigas*), and the blue mussel (*Mytilus* spp., hereafter *Mytilus*). There is an increasing need for efficient methods to monitor these species as flat oysters and blue mussels are declining in their native ranges (Baden et al., 2021; Fariñas‐Franco et al., 2018; Sorte et al., 2017; Thurstan et al., 2013), and the Pacific oyster has emerged as an invasive bivalve (Ruesink et al., 2005) leading to concerns about competition with native bivalves. We address three research questions relating to the aforementioned knowledge gaps. First, to quantify the error of towed video in estimating abundance, we compare the number of individuals detected in the video to the “true” number of individuals present, determined by manually surveying an entire transect. As reliability also is related to variation within and between the observers analyzing the videos, we estimate the effect of observer variation. Second, we compare the reliability and efficiency of the video method to a method that subsamples a fixed area with quadrats. To evaluate the efficiency of the two methods, we compare the time used for each method in relation to the time it takes to sample the same area manually. In addition, we discuss how these methods have been applied for monitoring at the request of local resource managers in Scandinavia.

## 2 MATERIALS AND METHODS

### 2.1 Study area and sampling design

The methods were evaluated on data collected from three sites along the Norwegian south coast (58.267494° N, 8.519409° E; 58.200711° N, 8.297240° E; 58.129141° N, 8.147386° E) (Figure 2b). The sites were selected based on the criteria that they had all three species of interest existing at overlapping depths and varying densities of bivalves. At each site, three 20‐m transects were defined by placing a rope ladder on the seafloor between 0.5 and 1 m depth, forming 10 sections and a total area of 11 m^2^ (Figure 2c). The nine transects together encompassed a wide range of substrates including soft sand, gravel, and rocky substrates. First, to estimate bivalve density based on video, we filmed the transect by slowly pulling a metal sled (Figure 2a) mounted with a GoPro Hero 8 camera over the rope ladder (hereafter video method). Second, to estimate bivalve density based on quadrat sampling, we placed three 0.25 m^2^ metal squares within the rope ladder (Figure 2c) and waded into the water to count the number of individual bivalves and quantify the algae cover in each square (hereafter quadrat method). Lastly, to determine the “true” density of the transect, we examined seafloor covered by the entire rope ladder (Figure 2c) by counting the number of individual bivalves and quantifying the algae cover in each of the 10 sections of the rope ladder (hereafter “true” density). To test if biological parameters affect the ability of observers to estimate the number of bivalves in videos, we noted if individual bivalves were living or not, if they were in clusters or solitary, and measured their shell dimensions. The species and living status of the bivalves were determined according to Table 1.

**TABLE 1:** Criteria for classifying the living status of bivalves and for distinguishing between the two oyster species (*Ostrea edulis* or *Magallana gigas*) during video analysis. Table adapted from Thorngren et al. (2017).

Subject	Category	Definition
Living or dead	Living	Distinct to moderate three‐dimensional shell structure
		Nuanced coloration
		Two visible shells with a small gap
		Dark edge/hollow in the sediment around the oyster
	Dead	White or bright shells
		Two visible shells with a large gap
	Not counted	Single shells and shell fragments
Species	*O*. *edulis*	Rounder and flatter than *M*. *gigas*
		Wavy edge that is evenly wavy
		Narrower filtration gap than *M*. *gigas*
		Thinner growth zone that is more evenly colored
	*M*. *gigas*	Large and uneven shape
		Larger filtration gap than *O*. *edulis*
		Growth zone sometimes striped with purple/dark stripes

### 2.2 Analysis of video material

The videos from each transect were split into 10 clips, one for each section (1.1 m^2^) of the rope ladder, using iMovie (Version 10.3.5) for Mac OSX. Each video clip was assigned a random ID to prevent observers from knowing from which site the video originated. Ten observers went through a 2‐h training course where they were taught to analyze the videos based on a pre‐defined set of criteria (see Table 1). To test if previous hands‐on experience with bivalves affects the ability of observers to estimate the number of bivalves in a video, we included five observers with, and five observers without, previous bivalve field experience. To compare the error of the video and quadrat method on a transect level, two experienced observers analyzed all 90 videos from the nine transects. To quantify interobserver variation, the remaining eight observers analyzed an identical subset of videos (*n* = 30). To quantify intraobserver variation, all observers repeated at least 10 of these videos randomly and “blindly” a second time. It would be ideal to have all observers analyze all 90 videos twice; however, due to video analysis being time‐consuming, we decided to analyze a subsample of the videos to maintain sufficient sample size while reducing the time used.

### 2.3 Statistical analyses

#### 2.3.1 Evaluation of towed video method

To estimate the difference between the measured density and the “true” density (hereafter error) and to investigate the causes of this error, we used the log ratio between the number of individuals detected in the video and in the rope ladder as the response, calculated as:

$$\log\left(\frac{\text{Number of individuals in video}+1}{\text{Number of individuals in rope ladder}+1}\right)$$

The log ratio was selected as the response variable instead of absolute or relative error because the two latter were highly right skewed, causing a lack of homogeneity. To account for non‐independence due to multiple observations of each video, we used linear mixed‐effects models with the unique ID assigned to each video as a random effect. We additionally included video ID nested within observer as a random effect to account for observers analyzing videos more than once. Models were fitted separately for each species using the *lme4* package (v1.1–34; Bates et al., 2015).

We first fit a null model for each species. The intercept of this model describes the overall error, regardless of the cause. This intercept would be zero if the number of individuals in the video is equal to the “true” number of individuals, while fewer or more individuals detected in the video would lead to a negative or positive intercept, respectively. The log ratio is, by definition, symmetric around zero, meaning that counting x too few individuals and x too many individuals in the video (compared to the “true” number of individuals) will lead to the same value, but with opposite sign. To investigate what is causing the error, we then fit a full model with all fixed effects expected to affect error, including algae cover, total number of bivalves, proportion of oysters in clusters, proportion of large and small oysters, and binary level of observer experience (Table 2). The cluster and size variables were species‐specific, meaning that the model for *O*. *edulis* included the proportion of *O*. *edulis* in clusters and the proportion of large/small *O*. *edulis* in each section, and so on. The large‐ and small‐size indices were determined by multiplying the size dimensions (length × width × height in mm) of all individuals found at all three sites and based on these, calculating the overall species‐specific lower‐ and upper‐size quartile. The proportion of individuals smaller than, and larger than, the lower and upper quartile in each rope ladder section was then calculated and used as the large‐ and small‐size index, respectively.

**TABLE 2:** An overview of the explanatory variables in the linear mixed‐effects models explaining observation error from the towed video method for each of the three species *Magallana gigas*, *Ostrea edulis*, and *Mytilus* spp.

Expl. variable	Description	Direction of error
Algae cover	Proportion of section covered in algae	Algae can obscure bivalves and lead to underestimation, especially for *M*. *gigas* that tends to live under macroalgae
Cluster index	Proportion of oysters in the section that are in a cluster	Sections with more individuals in clusters can lead to underestimation because you may not see all individuals in a cluster
Large size index	Proportion of large oysters in a section. Large oysters are defined as the upper quartile from the entire dataset of sizes	Sections with large oysters can lead to underestimation because they can obscure oysters underneath
Small size index	Proportion of small oysters in a section. Small oysters are defined as the lower quartile from the entire dataset of sizes	Sections with small oysters can lead to underestimation because they are more difficult to see with the video
Number of bivalves	Number of living and dead *M*. *gigas*, *O*. *edulis*, and *Mytilus* individuals found in a section	Higher abundances of bivalves can lead to underestimation because they are covering bivalves underneath
Observer experience	Factor with two levels: experienced or inexperienced with bivalves	Observers with less experience with bivalves are more likely to misclassify species or fail to detect bivalves than observers with experience

*Note*: The table describes the variables and hypothesizes the direction of error caused by them.

To be able to include cluster and size information as variables in the model, we excluded all videos where the species of interest was absent, since information about clusters and size is undefined if no individuals were present. This reduced the sample size from 30 videos to 27 for *M*. *gigas*, and to 21 for *O*. *edulis*, with a corresponding reduction from 440 ([8 observers × {30 videos + 10 repeat}] + [2 observers × {30 videos × 2 repeats}] = 440 total observations) video observations to 395 for *M*. *gigas*, and to 309 for *O*. *edulis* (Table 3). The full model for *Mytilus* did not include cluster or size variables, as there were so few individuals in clusters and they were similar in size, and thus the sample size for this species was maintained at 440 observations.

**TABLE 3:** Fixed effects from a linear mixed‐effects model explaining observation error for three species of coastal bivalves (*Magallana gigas*, *Ostrea edulis*, and *Mytilus* spp.).

Species	Model (*n*)	Predictors	Estimate	SE	*t*	95% CI	*p*
*Magallana gigas*	Null (440)	Intercept	−0.30	0.12	−2.42	[−0.55, −0.5]	
	Null (395)	Intercept	−0.36	0.12	−3.00	[−0.59, −0.12]	
	Full (395)	Intercept	−0.23	0.13	−1.77	[−0.47, 0.03]	.11
		Algae cover	0.06	0.13	0.50	[−0.18, 0.32]	.59
		Cluster	−0.21	0.11	−1.94	[−0.43, 0.003]	.06
		Large oysters	−0.04	0.11	−0.38	[−0.26, 0.17]	.70
		Small oysters	−0.19	0.10	−1.87	[−0.39, 0.01]	.06
		Number of bivalves	−0.23	0.11	−2.08	[−0.45, −0.004]	.04
		Inexperienced	−0.11	0.15	−0.76	[−0.40, 0.17]	.51
	Min. ad. (395)	Intercept	−0.29	0.10	−2.87	[−0.48, −0.09]	<.01
		Cluster	−0.18	0.08	−2.15	[−0.34, −0.02]	.05
		Small oysters	−0.18	0.09	−1.91	[−0.35, −0.004]	.08
		Number of bivalves	−0.23	0.10	−2.39	[−0.42, −0.05]	.03
*Ostrea edulis*	Null (440)	Intercept	−0.33	0.14	−2.24	[−0.60, −0.03]	
	Null (309)	Intercept	−0.70	0.13	−5.40	[−0.97, −0.44]	
	Full (309)	Intercept	−0.70	0.18	−3.92	[−1.05, −0.34]	<.01
		Algae cover	0.05	0.13	0.42	[−0.19, 0.30]	.65
		Cluster	−0.37	0.19	−1.98	[−0.72, −0.02]	.07
		Large oysters	0.04	0.21	0.20	[−0.38, 0.46]	.84
		Small oysters	0.03	0.24	0.13	[−0.44, 0.48]	.90
		Number of bivalves	−0.13	0.14	−0.87	[−0.40, 0.15]	.42
		Inexperienced	−0.05	0.20	−0.25	[−0.45, 0.34]	.82
	Min. ad. (309)	Intercept	−0.77	0.13	−5.99	[−1.02, −0.52]	<.01
		Cluster	−0.31	0.15	−2.15	[−0.59, −0.03]	.04
*Mytilus*	Null (440)	Intercept	−0.20	0.13	−1.60	[−0.44, 0.05]	
	Full (440)	Intercept	−0.38	0.11	−3.37	[−0.60, −0.16]	<.01
		Algae cover	−0.02	0.13	−0.19	[−0.28, 0.23]	.85
		Number of bivalves	−0.19	0.12	−1.58	[−0.43, 0.04]	.13
		Inexperienced	0.42	0.08	5.26	[0.25, 0.57]	<.01
	Min. ad. (440)	Intercept	−0.41	0.11	−3.64	[−0.63, −0.19]	<.01
		Inexperienced	0.42	0.08	5.26	[0.26, 0.57]	<.01

*Note*: The table gives an overview of the models fit for each species, the number of observations included in each model (*n*), the fixed effects included in each model, the estimate, standard error, *t*‐value, 95% confidence interval, and *p*‐value.

To obtain a minimum adequate model, we used stepwise backward model selection, using the likelihood ratio test, starting with the full model. To be able to reliably compare the performance of the null, full, and minimum adequate models, we needed to compare models based on the same dataset. We therefore fit another null model using the same reduced datasets for *M*. *gigas* and *O*. *edulis* as was used for the full and minimum adequate model (see Table A1). The inference from the null model can be used, in future studies, to compensate for any overall observer error, which can be necessary as information about in situ habitat characteristics in such circumstances often is unavailable. The minimum adequate model shows which factors, and to what extent these, are causing observation error. By comparing the null model performance with the performance of the other models, we can investigate how vital information on habitat/site characteristics is in order to get reliable density estimates.

To estimate the variation in error explained by the random effects, we carried out a variance component analysis. To estimate confidence intervals and *p*‐values of the model terms, we used parametric bootstrapping (*B* = 1000) in the *lme4* (v1.1–34; Bates et al., 2015) and *lmeresampler* packages (v0.2.4; Loy et al., 2023), respectively. All statistical analyses were performed using the R programming language (v4.3.1; R Core Team, 2023).

#### 2.3.2 Comparison of towed video and quadrat method

To compare the reliability of the video and quadrat method, we used a paired t‐test to compare the log ratio in density estimated from each method respectively for the nine transects to the “true” density of the transect (*rstatix* package v0.7.2; Kassambara, 2023). To estimate the classification accuracy for each method, we created a confusion matrix cross tabulating the actual occurrence of each species in a transect and their observed occurrence as determined by the video or quadrat method, respectively (Table A2.1). We created confusion matrices for detecting both presence‐absence and high density or not high density (high density defined as ≥1 individual/m^2^ in Bergström et al., 2021, 2022) of bivalves as these methods have previously been used for detecting both of these response variables (Table A2.2). Based on the confusion matrix, we derived the following three metrics; (1) the correct classification rate, which is the proportion of correct classifications in which a method detects a species as present when it is truly present and absent when truly absent, (2) the false absence rate which refers to when a method fails to detect the presence of a species, and (3) false presence rate which refers to when a method falsely detects a species is present (Table A2.3). For all classification accuracy estimation, we only used the video observation from the most experienced observer and used the average of the two repeats of that observer.

### 2.4 Application of methods

To gain an overview of how the video and quadrat methods have been used for surveying epibenthic bivalves, we reviewed the literature and contacted our scientific networks. Specifically, we were searching for information about where the video and quadrat methods have been applied (Figure 2b), the time used to survey a site in the field using these methods, and the time used for the video analysis. To evaluate the efficiency of these methods, we calculated the average time used to survey a transect using each method and compared this to the time it took for us to survey an entire transect manually in the rope ladder. We decided to base the time estimates for the video and quadrat methods on real applications of the methods rather than the experimental design of this project to have a more reliable estimate based on a larger sample size. An extended description of the two methods is provided in the Appendix 1 (see section A3 and A4).

## 3 RESULTS

### 3.1 General observations

**FIGURE 3:**
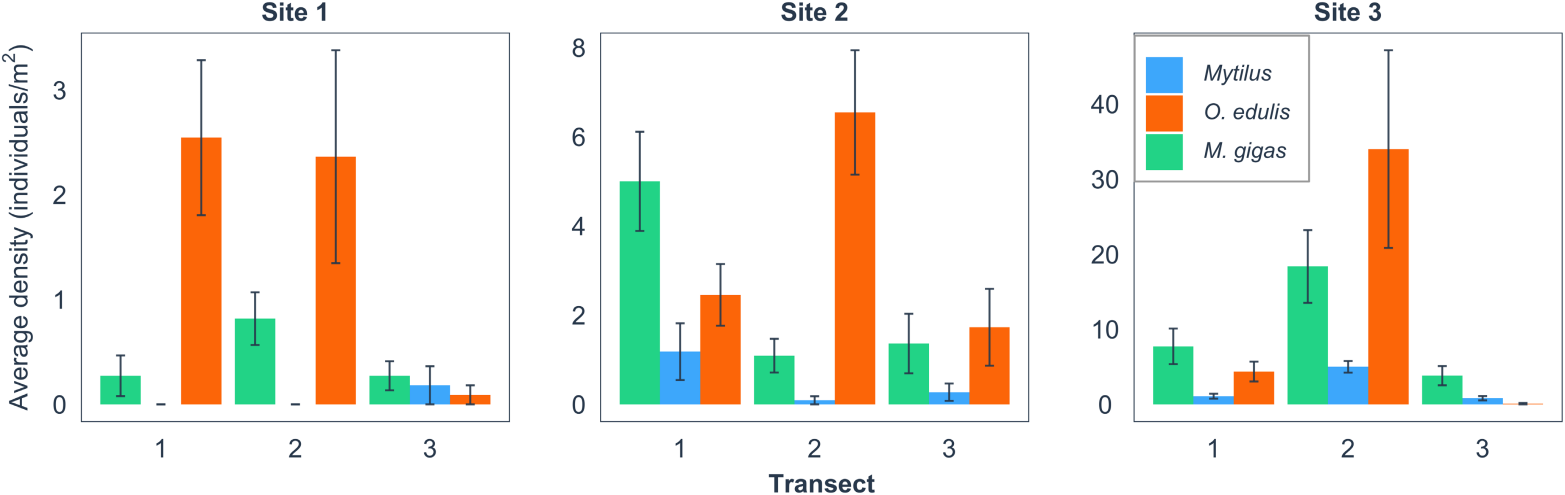
The average density (number of individuals/m^2^) of *Magallana gigas*, *Ostrea edulis*, and *Mytilus* in the three transects at each site. Error bars represent the standard error of the 10 sections (1.1 m^2^) in each transect. Note the different scales of the y‐axes.

A total of 596 living *O*. *edulis*, 426 living *M*. *gigas*, and 94 living *Mytilus* individuals were found, based on in situ observations of the nine transects at the three study sites. The maximum density found was 146.4 individuals/m^2^ for *O*. *edulis*, 48.2 individuals/m^2^ for *M*. *gigas*, and 7.3 individuals/m^2^ for *Mytilus*. In general, site one had the lowest density of all three species, site two had intermediate densities, and site three had the highest densities (Figure 3).

### 3.2 Evaluation of towed video method

**FIGURE 4:**
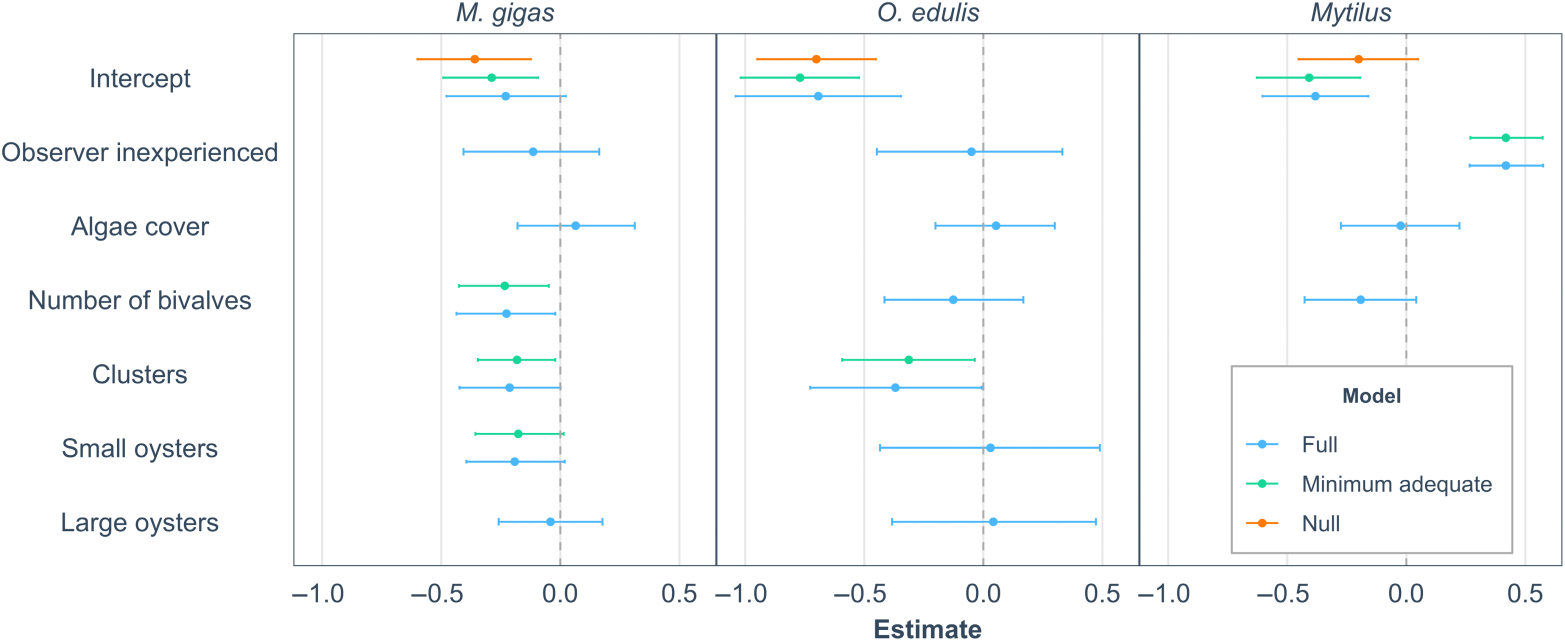
Model estimates and their 95% bootstrapped confidence intervals for each term included in the null model, full model, and minimum adequate model explaining observation error for each of the three species *Magallana gigas*, *Ostrea edulis*, and *Mytilus* spp. Negative estimates represent that the predictor variable of interest causes underestimation and vice‐versa.

The null models showed that the video analyses underestimated the abundance of *M*. *gigas*, *O*. *edulis*, and *Mytilus* by 23%, 24%, and 16%, respectively (Table 3). In the minimum adequate model, smaller individuals, and higher densities of bivalves in videos led to more underestimation of *M*. *gigas* (Table 3 and Figure 4). For both oyster species, increasing proportion of clusters led to increased underestimation (Table 3 and Figure 4). Finally, for *Mytilus*, observers without previous experience were more likely to overestimate than those who had more experience with bivalves (Table 3 and Figure 4).

**FIGURE 5:**
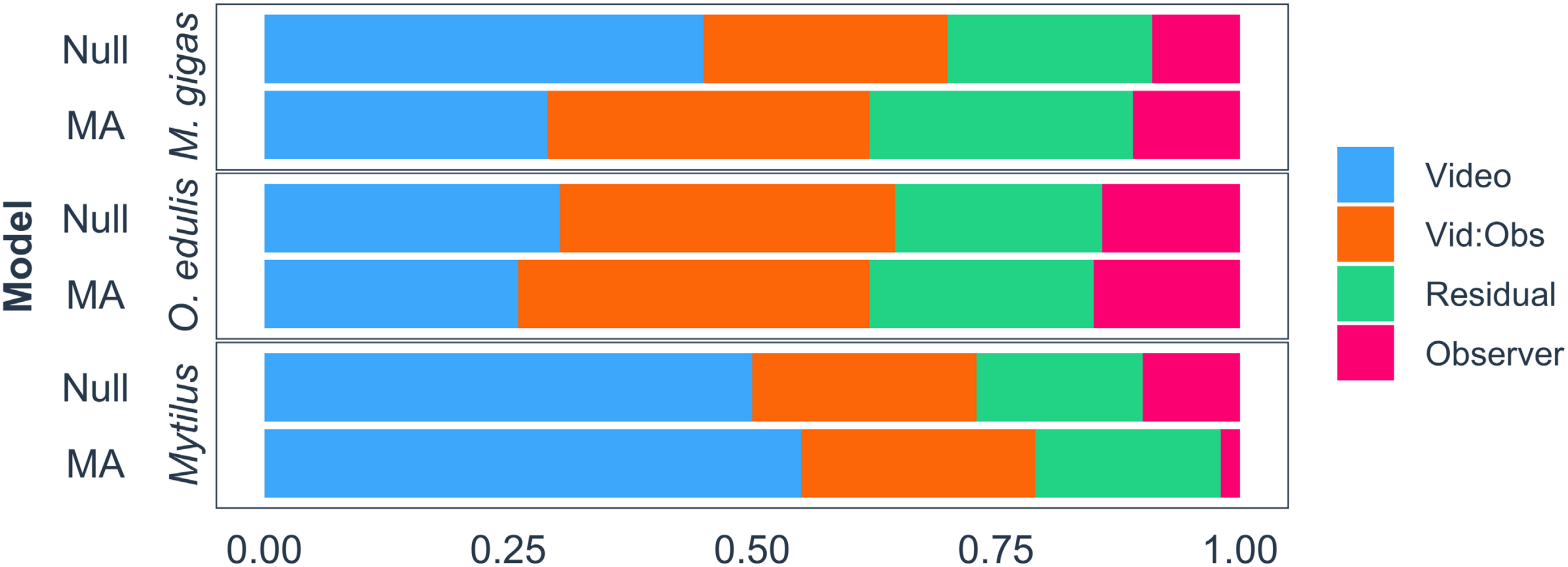
The percentage of total variance of observation error of coastal bivalves (*Magallana gigas*, *Ostrea edulis*, and *Mytilus* spp.) explained by video, observer nested within video (Vid:Obs), and observer, from a linear mixed‐effects model. Null model contains only random effects, and minimum adequate model (MA) contains random effects and remaining fixed effects after model selection.

For *M*. *gigas*, differences between videos contributed most to the total variability in the null model (45%). After adding fixed effects, the differences within observers from repeated measurements contributed slightly more to the total variance (33%) than differences between videos (29%). For *O*. *edulis*, differences within observers contributed most to the total variability in both the null (34%) and minimum adequate model (36%). The second largest component for *O*. *edulis* was the variability between videos, contributing 30% and 26% to the null and minimum adequate model, respectively. For *Mytilus*, the differences between videos were the dominating factor contributing to 50% and 55% of the variance in the null and minimum adequate models, respectively. For all three species, the differences between observers contributed least to the total variance in both the null (9%, 14%, and 10% for *M*. *gigas*, *O*. *edulis*, and *Mytilus*, respectively) and minimum adequate models (11%, 15%, and 2%, respectively; Figure 5).

### 3.3 Comparison of towed video and quadrat method

**FIGURE 6:**
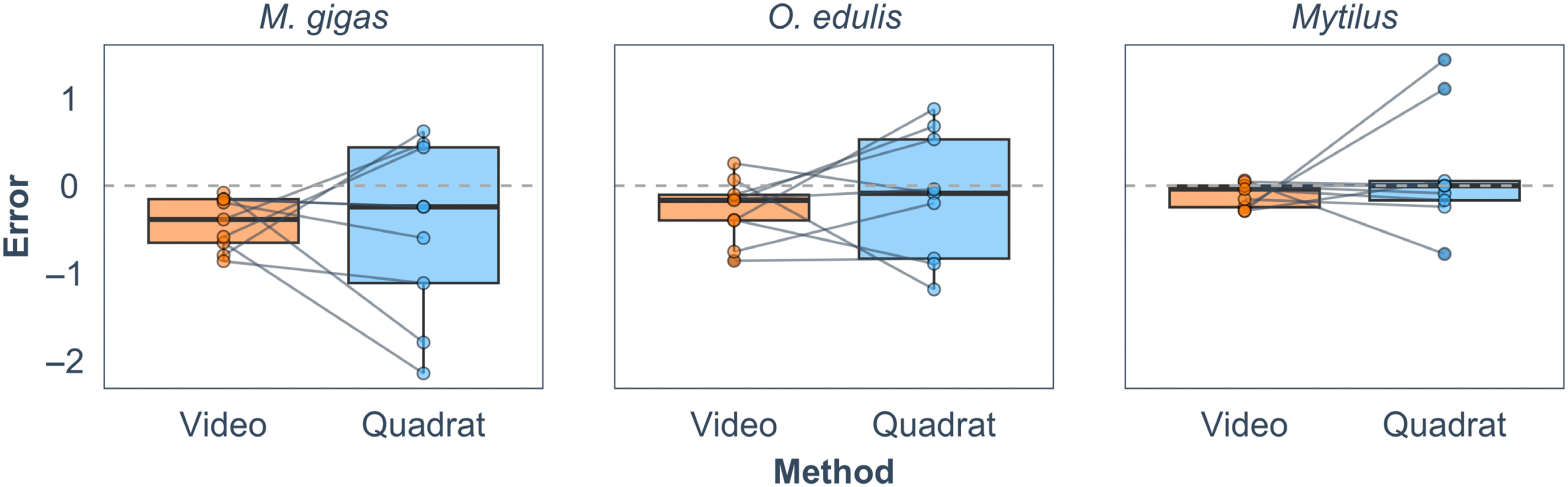
The observation error from the video and quadrat method for *Magallana gigas*, *Ostrea edulis*, and *Mytilus* spp. Positive values indicate overestimation, and negative values indicate underestimation. Points connected by lines indicate paired methods within transects (*n* = 9).

There was no statistically significant difference between the error in density estimated by the video method compared to the quadrat method for all three species (paired *t*‐test all with df = 8, *M*. *gigas*: *t* = 0.23, *p* = .83, *O*. *edulis*: *t* = −0.58, *p* = .58, and *Mytilus*: *t* = −0.93, *p* = .38; Figure 6). The average error was lower when using the video method compared to the quadrat method for *M*. *gigas* (−0.43 ± 0.30 and − 0.51 ± 1.00, respectively) and *Mytilus* (−0.10 ± 0.14 and − 0.15 ± 0.69, respectively). For *O*. *edulis*, the average error was higher when using the video method (−0.29 ± 0.36) than the quadrat method (−0.13 ± 0.73). The effect size for the difference between methods was negligible for *M*. *gigas* and *O*. *edulis* (*d =* 0.08 and −0.19, respectively) and small for *Mytilus* (*d* = *−*0.31).

The video method classified presences correctly more often than the quadrat method for all three species (Table 4). For detecting high densities (≥1 individual/m^2^), the video and quadrat method performed equally well for *M*. *gigas*, and video outperformed the quadrat method for *O*. *edulis* and *Mytilus* (Table 4). In transects where *M*. *gigas* and *Mytilus* were present, the quadrat method failed to detect their presence 56% and 57% of the time, respectively (Table 4). In contrast, the video method detected the presence of *M*. *gigas* and *Mytilus* in all transects where they were present (Table 4). The video method falsely detected *Mytilus* as present in transects when they were absent 50% of the time (Table 4).

**TABLE 4:** An overview of the classification accuracy metrics (correct classification rate, false absence rate, and false presence rate) calculated for the quadrat method and video method for detecting presence/absence and high densities (≥1 individual/m^2^) of *Magallana gigas*, *Ostrea edulis*, and *Mytilus* in each of nine investigated transects.

Species	Detecting	Method	Correct class	False absence	False presence
*Magallana gigas*	Presence/absence	Quadrat	0.44	0.56	NA
		Video	1	0	NA
	High density	Quadrat	0.78	0.33	0
		Video	0.78	0.33	0
*Ostrea edulis*	Presence/absence	Quadrat	0.78	0.22	NA
		Video	0.89	0.11	NA
	High density	Quadrat	0.78	0.14	0.5
		Video	0.89	0.14	0
*Mytilus*	Presence/absence	Quadrat	0.56	0.57	0
		Video	0.89	0	0.5
	High density	Quadrat	0.78	0.5	0.14
		Video	0.89	0.33	0

### 3.4 Application of methods

The video method has been used to survey epibenthic bivalves at 1466 sites, and the quadrat method has been used at 333 sites (Figure 2b). The time used was documented for the video method at 502 sites, for the video analysis at 81 sites, and for quadrat method at 177 sites (Table A5). It took more time on average to survey one site using the quadrat method (0.31 ± 0.11 h) than to record by towed video (0.12 ± 0.05 h); however, the following video analysis was time‐consuming (0.40 ± 0.25 h). Despite this, both methods are substantially more efficient than surveying the entire transect manually (1.88 ± 1.30 h) as we did in the rope ladder.

## 4 DISCUSSION

### 4.1 Evaluation of towed video method

This study tested the reliability of towed video as a method for estimating the abundance of ecologically important coastal epibenthic bivalves. By comparing the number of bivalves detected in videos to data collected in situ, we found higher error for the two oyster species than for *Mytilus*. Our findings that towed video underestimated by 24% for *O*. *edulis* are similar to a previous study that found video to underestimate by 20% for *O*. *edulis* (Thorngren et al., 2017). We found that the causes of underestimation for the two oyster species were biological parameters such as bivalves grouped in clusters, large amounts of small individuals, and generally higher abundances of bivalves. Although *Mytilus* were also underestimated overall, for this species observer experience was important with inexperienced observers overestimating and experienced observers underestimating. These findings imply that abundance estimates for oysters from video can only be partially corrected as some error cannot be accounted for without having information about individual size and the proportion of individuals in clusters. To an extent, some of this information can be extracted from the videos. For example, length measurements have been obtained from towed video for other epibenthic organisms (Ruhl, 2007), but the tendency for *M*. *gigas* to form reefs may make it challenging to obtain reliable length estimates at high densities.

Many studies have shown that underwater visual methods are prone to observer errors (Benedetti‐Cecchi et al., 1996; Couch et al., 2021; Thompson & Mapstone, 1997) and that observer experience affects the precision of data collected (Bernard et al., 2013; Williams et al., 2006). As the analysis of underwater imagery is also a visual method, it is likely that observer error exists; however, few studies have investigated this (but see Ninio et al., 2003; Thorngren et al., 2017). To our knowledge, this is the first study to investigate the effect of both observer variation and observer experience on error in the analysis of underwater imagery. We found that within‐observer differences contributed more to variation in error than between‐observer differences. This suggests that it would be an advantage to recruit multiple observers for the analysis of towed video footage. Furthermore, we found that inexperienced observers overestimated *Mytilus* compared to experienced observers, who underestimated. The videos with the greatest overestimation of *Mytilus* had many dead mussel shells, and it would be valuable to investigate if living and dead bivalves can be differentiated through image color and texture feature extractions as done for other species by Marcos et al. (2008).

### 4.2 Comparison of towed video and quadrat method

We found that the amount and direction of error from the video and quadrat method were comparable. This suggests that these two methods can be used in combination to provide full depth coverage of the littoral zone. While the videos only give information about abundance and substrate type, the quadrat method provides detailed information of individual size, mortality, and recruitment. The video method has a lower detection of smaller individuals and oysters in clusters compared to the quadrat method. However, the variation in error from the quadrat method is larger than that of the video method, probably due to a small area covered and a non‐homogenous distribution of the three species. To reduce variation from the quadrat method, the number of squares could be increased, but at the expense of increased time at each site or reduced number of sites. The solution to this trade‐off is case‐specific and depends on the management objectives of the survey. If the purpose is to detect an invasive species in the expansion phase, when they are not widespread, it is advantageous to increase the area sampled to minimize false zeros. This is supported by our findings that the quadrat method has a higher false absence rate than the video method which covers more ground.

The video method had a lower false absence rate for all three species, but it had a higher false presence rate for *Mytilus*, detecting presence in half of the videos where they were absent. In all cases where *Mytilus* was falsely detected in the videos, *Mytilus* shell fragments were common, so shell fragments both cause overestimation when *Mytilus* is present and false positives when it is not. This suggests that video methods are more suitable for mapping high densities of *Mytilus* rather than detecting presence‐absence. Another dimension that should be considered is the suitability of these methods in relation to the habitat preferences of the target species. *Mytilus*, for example, are often found on vertical or oblique cliffs where it is not feasible to survey with towed video. In such habitats, remotely operated underwater vehicles or underwater visual census are more suitable methods than seabed‐towed video (Esagholian Khoygane, 2021; Nevstad, 2022; Strohmeier et al., 2022). In cases where protecting the seabed from disturbance is important or where bottom conditions make towing a sled difficult, a towed video rig hovering above the seabed may be more suitable, albeit more expensive (Sheehan et al., 2016). Additional challenges with underwater video methods can be turbidity or the suspension of sediments, both of which can blur the visibility; swells making operation of equipment difficult and the draught of the vessel limiting access to shallow waters.

### 4.3 Synthesis and applications

In the face of global environmental change and biodiversity loss, it is essential to have reliable and efficient tools for mapping and monitoring species over large spatial scales to establish population baselines and assess trends over time. The video and quadrat methods that we have tested provide an advantage over traditional sampling methods (e.g., fishing, dredging) because they are less destructive, and the videos can be reused for other monitoring purposes. These methods have been used to map new, and monitor existing, epibenthic macrofauna at the request of local and national management bodies in Scandinavia. For example, towed video has been used to establish population baselines for a threatened species (Thorngren et al., 2019), to evaluate the overlap between native and invasive species (Bergström et al., 2021; Reamon et al., 2021), and to map invasive species in different stages of the invasion process (Reamon et al., 2021, 2022). For effective management of invasive species, early action is the most effective strategy (Simberloff et al., 2013). In Scandinavia, one of the current management actions for early stages of the invasion of Pacific oysters is to remove them to prevent further range expansion (Postmyr, 2016). To guide management towards sites to prioritize, the video and quadrat methods have been used in combination to map the abundance of Pacific oysters and identify hotspots (Reamon et al., 2022).

We found that the video and quadrat methods are time efficient as it took substantially less time to sample one site in the field using these methods than manually surveying a full transect. Even when we accounted for the time it took to analyze the videos, this method still took less than a third of the time of surveying the entire transect manually. To further improve the efficiency of the video processing, it could be valuable to implement video recognition tools and/or underwater hyperspectral imaging, which has been used for mapping benthic habitats (Montes‐Herrera et al., 2021) including oyster reefs (Barillé et al., 2017; Nielsen et al., 2022). A simpler option to improve the efficiency of the video method would be to use % coverage of bivalves as a metric rather than the number of individuals, specifically where there are high densities and counting the number of individuals is time‐consuming.

Our study shows that the video and quadrat methods are efficient and are therefore useful for mapping over large spatial scales. We found that both methods are reliable for estimating the abundance of three keystone macrofaunal species, suggesting that they can also be useful for other similar species as well. However, we show that the errors are species‐specific, and the method should therefore not be applied to new species without validation. The two methods used in combination provide reliable data across the full depth range of the littoral zone as the quadrat method can be used for sampling intertidal waters and the video method can be used for sampling subtidal waters. We demonstrate case studies where these methods have been used to map invasive species, establish population baselines of threatened species, and investigate the overlap between invasive and native species. Based on these case studies and our findings that these methods are efficient and reliable, we propose that the methods can contribute to improve both scientific knowledge and conservation outcomes.

## AUTHOR CONTRIBUTIONS

**Molly Reamon:** Conceptualization (lead); data curation (lead); formal analysis (lead); investigation (equal); methodology (lead); visualization (lead); writing – original draft (lead); writing – review and editing (equal). **Johanna B. Marcussen:** Conceptualization (supporting); data curation (supporting); formal analysis (supporting); investigation (equal); writing – original draft (supporting); writing – review and editing (equal). **Ane T. Laugen:** Conceptualization (supporting); formal analysis (supporting); funding acquisition (lead); investigation (supporting); resources (lead); supervision (equal); writing – review and editing (equal). **Lars M. Korslund:** Conceptualization (supporting); formal analysis (supporting); investigation (supporting); supervision (equal); writing – review and editing (equal).

## CONFLICT OF INTEREST STATEMENT

The authors have no conflicts of interest to declare.

## Data Availability

All data and code are available through the Open Science Framework https://osf.io/9j2hx/?view_only=9f148e840f894890847d0b6c3f3b31f0

## APPENDIX 1

### A1: MODEL COMPARISON THROUGH AIC

**TABLE A1:** Comparison of the linear mixed‐effects models with log ratio between video observation and field observation as response variable for three species of coastal bivalves (*Magallana gigas*, *Ostrea edulis*, and *Mytilus* spp.).

Species	Model	Fixed effects	Df	AIC	ΔAIC
*Magallana gigas*	Null	None	5	621.27	3.83
	Full	Algae cover, proportion of *M*. *gigas* in clusters, proportion of small *M*. *gigas*, proportion of large *M*. *gigas*, number of bivalves, and level of observer experience	11	629.70	12.26
	Minimum adequate	Proportion of *M*. *gigas* in clusters, proportion of small *M*. *gigas*, and number of bivalves	8	617.44	0
*Ostrea edulis*	Null	None	5	532.06	0.31
	Full	Algae cover, proportion of *O*. *eduli*s in clusters, proportion of small *O*. *edulis*, proportion of large *O*. *edulis*, number of bivalves, and level of observer experience	11	548.40	16.65
	Minimum adequate	Proportion of *O*. *edulis* in clusters	6	531.75	0
*Mytilus*	Null	None	5	654.83	8.28
	Full	Algae cover, number of bivalves, level of observer experience	8	652.77	6.22
	Minimum adequate	Level of observer experience	6	646.55	0

*Note*: The random effects in all models were “Video ID” and “Video ID” nested within “Observer”. The table gives an overview of fixed effects included in each model, degrees of freedom, AIC values, and the difference in AIC values between the null, full, and minimum adequate models for each species. For *M*. *gigas* and *O*. *edulis*, models were fit only with data from videos where the species of interest was present to be able to include cluster and size dimensions.

### A2: CONFUSION MATRICES AND CLASSIFICATION ACCURACY METRICS

**TABLE A2.1:** Two‐way confusion matrix for comparing the observed value (presence/absence of a species observed based on the quadrat or video method) to the true value (presence/absence of a species in the rope ladder determined in the field). Several metrics can be calculated based on the matrix that is described in Table A2.3.

		True value (rope ladder)		
		Present (1)	Absent (0)	Total
Observed value (from quadrat or video method)	Present (1)	TP true presence	FP false presence	TP + FP
	Absent (0)	FA false absence	TA true absence	FA + TA
	Total	TP = FA	FP + TA	*N* Total number of observations

*Note*: Table adapted from Guisan et al. (2017).

**TABLE A2.2:** Confusion matrices for three species of coastal bivalves (*Magallana gigas*, *Ostrea edulis*, and *Mytilus* spp.), for each method (square and video method), and for each type of occurrence (presence/absence or high densities), resulting in 12 matrices.

*Magallana gigas*			Quadrat		Video	
			Present	Absent	Present	Absent
Rope ladder (“truth”)	Presence/absence	Present	4 (44.4)	5 (55.6)	9 (100)	0
		Absent	0	0	0	0
	High density (≥1 individual/m^2^)	Present	4 (44.4)	2 (22.2)	4 (44.4)	2 (22.2)
		Absent	0	3 (33.3)	0	3 (33.3)

*Ostrea edulis*			Quadrat		Video	
			Present	Absent	Present	Absent
Rope ladder (“truth”)	Presence/absence	Present	7 (77.8)	2 (22.2)	8 (88.9)	1 (11.1)
		Absent	0	0	0	0
	High density (≥1 individual/m^2^)	Present	6 (66.7)	1 (11.1)	6 (66.7)	1 (11.1)
		Absent	1 (11.1)	1 (11.1)	0	2 (22.2)

*Mytilus*			Quadrat		Video	
			Present	Absent	Present	Absent
Rope ladder (“truth”)	Presence/absence	Present	3 (33.3)	4 (44.4)	7 (77.8)	0
		Absent	0	2 (22.2)	1 (11.1)	1 (11.1)
	High density (≥1 individual/m^2^)	Present	1 (11.1)	1 (11.1)	2 (22.2)	1 (11.1)
		Absent	1 (11.1)	6 (66.7)	0	6 (66.7)

**TABLE A2.3:** Classification accuracy metrics derived from the confusion matrix.

Metric	Description	Formula
Correct classification rate	Percentage of correct classifications (presences and absences)	(TP + TA)/N
False absence rate	Percent of presences falsely classified	FA/(TP + FA)
False presence rate	Percentage of absences falsely classified	FP/(FP + TA)

*Note*: Table adapted from Guisan et al. (2017).

Abbreviations: FA, false absence; FP, false presence; *N*, number of observations; TA, true absence; TP, true presence.

### A3: Extended description of quadrat method

Modified from Mortensen et al. (2022).

Equipment:

- GPS for recording waypoints.
- 40‐m rope with markings at 10, 20, and 30 m.
- Squares that are 0.5 × 0.5 m and 0.25 × 0.25 m.
- Measuring stick.
- Aquascope.

Surveying methods:

1. Upon arrival, check if it is possible to survey the site. If not, note the substrate type and move to a nearby area with the same type of substrate. Choose an alternative location before checking for oysters to avoid bias.
2. Check the water level at the site:
  - In Sweden: https://www.smhi.se/vader/prognoser/vattenstand‐och‐vagor/
  - In Norway: https://www.kartverket.no/til‐sjos/se‐havniva
  - In Denmark: https://www.dmi.dk/malinger‐seneste‐24‐timer
3. For the 40‐m transect, follow the shoreline, and place the first square to the middle depth (0.25 m), then at every tenth meter place the square in the following order: deep (0.25–0.5 m); middle (0.25 m); shallow (0–0.25 m); middle (0. 25 m). Adjust all depths to current water level—see Table A3. Use a 0.5 × 0.5 m square unless there is a particularly high number of mussels or oysters, then use the small square (0.25 × 0.25 m).

**TABLE A3:** How to adjust positioning of the transect line according to current water level when aiming to survey at 0.25 m in normal water level.

If current water level… (cm)	Place the rope at this depth
+20	0.45 m
−20	0.05 m
<25	At shoreline
>50	Place “deep” squares (2 and 5) on the rope

4 In each square, record the following:
  - Site number.
  - Coordinates (WGS84, DD) for each square (center position).
  - Date.
  - Time.
  - Square number.
  - Square size.
  - Depths: (i) current water level according to your national water level forecast, (ii) actual depth at the time of surveying, and (iii) corrected depth.
  - Percentage coverage of all *Mytilus*, *M*. *gigas*, *O*. *edulis*, and of living *Mytilus*, *M*. *gigas*, *O*. *edulis*.
  - Number of live *Mytilus*, *M*. *gigas*, *O*. *edulis* (Only count mussels and oysters >15 mm, that is, disregard recruits from this year).
  - Percentage of each substrate category: Soft, Sand, Gravel, Shell hash, Shell, Rock, Cliff (the total should add up to 100%).
  - Percentage coverage of filamentous or macroalgae. Count the percentage of algae that covers the square when in normal water level.
  - Percentage cover of *Zostera* sp.

Definitions of substrate types:

Soft: Mud‐like substrate usually coarser and more humid than sand.

Sand: Granular material composed of finely divided rocks and minerals, finer than gravel and coarser than silt.

Gravel: Loose aggregation of rock fragments ranging from 2 mm to 5 cm.

Shell: Loose shell accumulations with a median particle size of 2 mm to <64 mm. Shells may be broken or whole.

Rock: Rocks larger than 5 cm loosely aggregated within the square.

Cliff: Mass of rock in an almost vertical position. Cannot be moved/part of mainland.

### A4: Extended description of towed video method

Modified from Mortensen et al. (2022).

Materials:

- Coordinates of randomly generated sites.
- GPS for registering coordinates of start and end of transect.
- Waterproof video camera (we use GoPro Hero 8).
- Extra SD memory cards.
- Extra camera batteries.
- Whiteboard and markers.
- Video sled.
- Rope longer than 50 m, attached to the front of the video sled.
- Boat with depth sonar.

Surveying methods:

- When you arrive to a site, assess the area with nautical charts, depth sonar, and look for warning signs that could indicate electrical cables or water pipes. Drive the boat over the area you intend to survey to check if it is possible to implement a 40‐m transect within the intended depth interval.
- Write the site name, date, and time on the whiteboard. Turn on the camera, start recording, and show the whiteboard to the camera before lowering the sled into the water while holding the rope. When the sled has reached the seafloor, take a waypoint on the GPS.
- Drag the sled behind the boat at a speed slower than 0.4 knots to ensure clear resolution of the videos. Keep an eye on the depth sonar and note the minimum‐ and maximum depth of the transect.
- After 40 m, take a new waypoint indicating the end of the transect and begin to pull the sled up.
- Before you leave the site, look quickly through the video to make sure the sled did not land upside‐down, remained on the seafloor throughout the entire transect, and that there is enough light to analyze the video.

Data to record for each transect:

- Unique site name.
- Date.
- Time at start and end of transect.
- Waypoints at start and end of transect.
- Depth (minimum and maximum depth of the transect).

After fieldwork

- Download the videos after each field day to make it easier to keep track of the files and to avoid losing data should something happen to the camera. Name the files so that the date and site name/number are included.
- Import waypoints from the GPS to your computer.

### A5: ESTIMATION OF TIME USED FOR EACH METHOD

**TABLE A5:** The average (mean ± SD) amount of time (in hours) it took to survey a 40‐m transect using the towed video method and the quadrat method compared to manually surveying the entire area as we did in the rope ladder.

Method	*n*	Hours/40‐m transect	Hours/m^2^	Area sampled (m^2^)
Towed video fieldwork	502	0.12 ± 0.05	0.003 ± 0.002	32
Towed video analysis	81	0.40 ± 0.25	0.01 ± 0.01	
Quadrat sampling	177	0.31 ± 0.11	0.25 ± 0.09	1.25
Rope ladder	9	1.88 ± 1.30	0.09 ± 0.06	11

*Note*: The time it took to survey with the towed video is split into the time used for fieldwork and video analysis. The table also shows the area (m^2^) that was sampled using each of the methods and amount of time (in hours) used per square meter.
